# SNP- and haplotype-based genome-wide association studies for growth, carcass, and meat quality traits in a Duroc multigenerational population

**DOI:** 10.1186/s12863-016-0368-3

**Published:** 2016-04-19

**Authors:** Shuji Sato, Yoshinobu Uemoto, Takashi Kikuchi, Sachiko Egawa, Kimiko Kohira, Tomomi Saito, Hironori Sakuma, Satoshi Miyashita, Shinji Arata, Takatoshi Kojima, Keiichi Suzuki

**Affiliations:** National Livestock Breeding Center, Nishigo, Fukushima 961-8511 Japan; Miyazaki Branch of National Livestock Breeding Center, Kobayashi, Miyazaki 886-0004 Japan; Graduate School of Agricultural Science, Tohoku University, Sendai, Miyagi 981-8555 Japan

**Keywords:** Duroc pigs, Haplotype-based GWAS, Known candidate genes, Production traits, SNP-based GWAS

## Abstract

**Background:**

The aim of the present study was to compare the power of single nucleotide polymorphism (SNP)-based genome-wide association study (GWAS) and haplotype-based GWAS for quantitative trait loci (QTL) detection, and to detect novel candidate genes affecting economically important traits in a purebred Duroc population comprising seven-generation pedigree. First, we performed a simulation analysis using real genotype data of this population to compare the power (based on the null hypothesis) of the two methods. We then performed GWAS using both methods and real phenotype data comprising 52 traits, which included growth, carcass, and meat quality traits.

**Results:**

In total, 836 animals were genotyped using the Illumina PorcineSNP60 BeadChip and 14 customized SNPs from regions of known candidate genes related to the traits of interest. The power of SNP-based GWAS was greater than that of haplotype-based GWAS in a simulation analysis. In real data analysis, a larger number of significant regions was obtained by SNP-based GWAS than by haplotype-based GWAS. For SNP-based GWAS, 23 genome-wide significant SNP regions were detected for 17 traits, and 120 genome-wide suggestive SNP regions were detected for 27 traits. For haplotype-based GWAS, 6 genome-wide significant SNP regions were detected for four traits, and 11 genome-wide suggestive SNP regions were detected for eight traits. All genome-wide significant SNP regions detected by haplotype-based GWAS were located in regions also detected by SNP-based GWAS. Four regions detected by SNP-based GWAS were significantly associated with multiple traits: on *Sus scrofa* chromosome (SSC) 1 at 304 Mb; and on SSC7 at 35–39 Mb, 41–42 Mb, and 103 Mb. The *vertnin* gene (*VRTN*) in particular, was located on SSC7 at 103 Mb and was significantly associated with vertebrae number and carcass lengths. Mapped QTL regions contain some candidate genes involved in skeletal formation (*FUBP3;* far upstream element binding protein 3) and fat deposition (*METTL3;* methyltransferase like 3).

**Conclusion:**

Our results show that a multigenerational pig population is useful for detecting QTL, which are typically segregated in a purebred population. In addition, a novel significant region could be detected by SNP-based GWAS as opposed to haplotype-based GWAS.

**Electronic supplementary material:**

The online version of this article (doi:10.1186/s12863-016-0368-3) contains supplementary material, which is available to authorized users.

## Background

Even before performance testing in pig breeding, single nucleotide polymorphisms (SNPs) associated with economically important traits (also referred to as quantitative trait loci [QTL]) can be useful in the selection of young animals. Several local animal experiment stations in Japan have performed closed-line breeding for more than five generations of purebred pigs [1, 2]. To improve these purebred populations using QTL information, segregation of QTL within a purebred population is necessary. One of the methods to detect segregation of QTL that affect economically important traits is genome-wide linkage-based QTL mapping in closed-line breeding populations as described by Uemoto et al. [3] and Soma et al. [4]. Using a purebred Duroc population comprised of multigenerational pedigree, these researchers detected several QTL and further fine mapping studies revealed significant SNPs in positional candidate genes, such as the stearoyl-CoA desaturase (*SCD*) gene for fatty acid composition [5] and the leptin receptor (*LEPR*) gene for fat accumulation [6]. These QTL can be used in the breeding management of purebred populations. Therefore, closed-line breeding population is useful to detect QTL.

Recent studies have reported genetic loci that are associated with complex traits using genome-wide association studies (GWAS) with high-density SNP arrays [7], and have demonstrated the power of GWAS to detect significant QTL in pig populations. This linkage disequilibrium (LD)-based QTL mapping with SNP array gives hope to the prospect of finding more QTL for economically important traits in pig populations. Linkage-based GWAS and LD-based GWAS can both detect significant SNPs in LD with common QTL variants that exert medium to high effects; however, LD-based GWAS permits finer mapping than linkage-based GWAS does [8]. Some studies have drawn comparisons between the methods employed for linkage-based GWAS and LD-based GWAS using SNP arrays in half-sib or F_2_ intercross pig populations [9–11], and found that the results of LD-based GWAS were generally consistent with those of linkage-based GWAS. However, to our knowledge, a comparison of both methods has not been performed in a purebred population comprising multigenerational pedigree. In addition, novel candidate regions might be detectable in such populations.

The purpose of the present study was (1) to compare the power between SNP-based GWAS and haplotype-based GWAS for QTL detection in simulation and real phenotype data analyses, and (2) to detect novel candidate genes affecting economically important traits. We used real genotype data of a purebred Duroc population comprising seven-generation pedigree in simulation analysis. These genotypes and 52 quantitative traits related to growth, carcass, and meat quality were used in real phenotype data analysis. We performed the linear mixed model association test at each SNP locus for LD-based GWAS (referred to as SNP-based GWAS), and at each haplotype locus for linkage-based GWAS (referred to as haplotype-based GWAS). Haplotypes were constructed based on pedigree and LD information [12].

## Methods

### Experimental animals

All procedures involving animals followed the Guidelines for the Care and Use of Laboratory Animals established by the National Livestock Breeding Center. A total of 836 Duroc purebred pigs at the National Livestock Breeding Center in Japan were used for GWAS. This population comprised one family from the first to the seventh generation; therefore, all individuals were related. Table 1 shows the number of animals by generation. The pigs had been selected on the basis of average daily gain (DG) from 30 to 105 kg of body weight, ultrasonically measured loin eye muscle area (LEA), backfat thickness (BF) at 105 kg weight, and intramuscular fat (IMF) content. The set breeding goals were as follows: 1100 g/d (for DG), 36 cm^2^ (for LEA), 2.6 cm (for BF), and 6 % (for IMF content). DG, LEA, and BF were measured in all pigs, and IMF content was measured in slaughtered sib-tested pigs. Boars (16) and gilts (22) were mated in the first generation, and 22 gilts from among their offspring and nine boars were then mated in the second generation. Pigs in the first and second generations were regarded as the base population. Closed-line breeding was performed from the third to the seventh generation, and phenotypes were determined in each generation. Pig in each generation were mated to account for inbreeding and to prevent rapid loss of genetic diversity, and a new generation was obtained each year. The inbreeding coefficient was estimated by the algorithm of Meuwissen and Luo [13], and the inbreeding coefficient of animals in each generation is shown in Additional file 1: Figure S1. Growth traits were measured in all pigs, and carcass and meat quality traits were measured in slaughtered sib-tested pigs. The selection method used in closed-line breeding was based on the methods of Suzuki et al. [1]. Breeding values of DG, LEA, BF, and IMF content were predicted by multi-trait animal model best liner unbiased prediction (BLUP), and aggregate breeding values were calculated by multiplying the relative economic weights by the estimated breeding value of each trait. The respective economic weights for DG, LEA, BF, and IMF content were assumed as 0.000, 0.882, −1.778, and 4.450, respectively. The aggregate breeding values were used as an indicator for selection in the breeding program.

**Table 1:** Population number by generation

	Male		Female		Total	
Generation	All	Sib-tested	All	Sib-tested	All	Sib-tested
1	16	0	22	0	38	0
2	19	10	40	11	59	21
3	42	19	47	10	89	29
4	67	31	86	26	153	57
5	73	40	86	21	159	61
6	71	39	86	27	157	66
7	80	44	101	30	181	74
Total	368	183	468	125	836	308

### Genotyping

Genomic DNA of 836 animals was extracted from ear tissue of 10-day-old pigs using proteinase K and the phenol method. Sample DNA was quantified and genotyped using the Illumina PorcineSNP60 BeadChip (Illumina, San Diego, CA, USA) according to the manufacturer protocol. Image data were analyzed with the iScan (Illumina) and genotype data were then called using the genotyping module contained in the GenomeStudio software (Illumina). Autosomal chromosomes were used and SNP quality control was assessed using the PLINK software [14]. The exclusion criteria for SNPs were minor allele frequency (MAF) <0.01, call rate <0.95, and Hardy-Weinberg equilibrium test <0.001. The exclusion criteria for animals was call rate <0.95.

Fourteen SNPs were chosen based on the current knowledge of physiological roles or the known candidate genes affecting pig meat quality and carcass traits, as shown in Table 2. These SNPs were genotyped by polymerase chain reaction-restriction fragment length polymorphism (PCR-RFLP), TaqMan SNP assay, or fragment length analysis. Marker information, such as associated production trait, PCR primers, reporters, and restriction enzymes for discriminating sequence variations are presented in Additional file 2: Table S1. Following quality control measures, the final data set included 831 individuals, which were genotyped at 38,128 SNPs (38,114 SNPs in the SNP array and 14 SNPs in the known candidate genes) and which were available for GWAS.

**Table 2:** Genetic markers associated with production trait

Chromosome	Gene symbol/SNP^a^	Gene name	Trait
1	*CYB5A*	*cytochrome b5 type A (microsomal)*	Boar taint
1	*MC4R*	*melanocortin-4 receptor*	Growth
2	*CAST*	*calpastatin*	Meat quality
4	*FABP4*	*fatty acid binding protein 4, adipocyte*	Fat
6	ALGA0113531	uncharacterized LOC102157459	Boar taint
6	*FTO*	*fat mass and obesity-associated*	Fat
6	*HFABP*	*heart fatty acid binding protein*	Fat
6	*LEPR*	*leptin receptor*	Fat
6	*PIK3C3*	*phosphoinositide-3-kinase, class 3*	Growth
7	*VRTN*	*vertnin*	Thoracic vertebrae number
8	*CCKAR*	*cholecystokinin type A receptor*	Growth
14	*GPR120*	*free fatty acid receptor 4*	Growth
14	*CYP2E1*	*cytochrome P450, family 2, subfamily E, polypeptide 1*	Boar taint

^a^Polymorphisms detailed information are shown in Additional file 2: Table S1

### LD information

We estimated LD coefficient (*r*^*2*^) values, which are a measure of LD, for all pairs of SNPs less than 10 Mb apart by PLINK software [14]. Average *r*^*2*^ values for a given intermarker distance, with marker distances grouped in 1 kb bins, were estimated in each autosome, and the average *r*^*2*^ values among chromosomes were then calculated. Average *r*^*2*^ values were then plotted against intermarker distance, as shown in Additional file 3: Figure S2. The results showed that moderate LD (the *r*^*2*^ value = 0.20) extended to about 1.0 Mb in this population. The threshold for useful LD was assumed to be *r*^*2*^ value ≧ 0.20 for the application of GWAS in this study, and the extent of LD (1.0 Mb) was applied for simulation study and QTL detection.

### SNP-based GWAS

We performed an SNP-based association study to detect significant SNPs. The adjusted phenotypes were first obtained by the following mixed model:

$$ \mathbf{y}=\mathbf{X}\mathbf{b}+\mathbf{Z}\mathbf{u}+\mathbf{e} $$

where **y** is the observation; **X** and **Z** are the design matrices for fixed and random effects, respectively; **b** represents sex (three classes; boar, barrow, and gilt) and generation (seven classes) effects as a fixed effect; this generation effect is the environmental effect at each generation; **u** represents polygenic effects distributed as *N*(0, **A***σ*_*u*_^2^); and **e** represents the residual effect distributed as *N*(0, **I***σ*_*e*_^2^). **A** is the numerator relationship matrix, *σ*_*u*_^2^ is polygenic variance, **I** is the identity matrix, and *σ*_*e*_^2^ is residual variance. The ASREML program [15] was used to estimate all effects, and the adjusted phenotypes (**y**_**adj**_) were then derived by

$$ {\mathbf{y}}_{\mathbf{adj}}=\mathbf{y}-\mathbf{X}\widehat{\mathbf{b}} $$

The adjusted phenotypes were then used as the dependent traits in a linear mixed model approach for each SNP:

$$ {\mathbf{y}}_{\mathbf{adj}}={\beta}_i{\mathbf{w}}_i+\mathbf{u}+\mathbf{e}{\mathbf{\hbox{'}}}_i $$

where *β*_*i*_ is the allele substitution effect; **w**_*i*_ is a vector of SNP genotypes (coded as 0, 1, or 2 for the homozygote, heterozygote, and the other homozygote, respectively); and **e’**_*i*_ is the random residual at the *i*-th SNP distributed as *N*(0, **I***σ*_*e* '_^2^). The regression coefficient and *p*-values tested by the Wald test were obtained by Genome-wide mixed-model association (GEMMA) software [16]. The proportion of phenotypic variance explained by the *i*-th SNP effects was calculated using the formula:

$$ Proportio{n}_i=\frac{2{p}_i\left(1-{p}_i\right){\beta}_i^2}{V_P} $$

where *p*_*i*_ is MAF of the *i*-th SNP and *V*_*P*_ is the adjusted phenotypic variance [17].

### Haplotype-based GWAS

Haplotype-based GWAS was performed on the basis of pedigree and LD information. Haplotypes were constructed using the hidden Markov model with DualPHASE [12], which assumes the number of ancestral haplotype states (K). A compromise value of K = 20 given the simulation considered [12] was used in the present study. The haplotype-based association analysis was then conducted using a linear mixed model using the GLASCOW software [18]. The method by GLASCOW software is composed of two steps: the residuals solved by linear mixed model without haplotype effect is firstly obtained, and the residuals are then used to test significance of association between the haplotype effect and the phenotype at each tested position. Adjusted phenotypes in SNP-based GWAS were used as the dependent traits in the model as follows:

$$ {\mathbf{y}}_{\mathbf{adj}}={\mathbf{1}}_{\mathbf{n}}\mu +{\mathbf{H}}_i{\mathbf{h}}_i+\mathbf{u}+\mathbf{e}{\mathbf{\hbox{'}}}_i $$

where **1**_**n**_ is a vector of *n* ones; *n* is the number of animals with phenotype; *μ* is the mean; **h**_i_ is a vector of haplotype random effect distributed as *N*(0, **I***σ*_*h*_^2^); **H**_i_ is the incidence matrix of haplotype genotypes for the individuals at the *i*-th haplotype locus; The element of **H**_i_ (*H*_*jk*_) is equal to the number of copies (from 0 to 2) of ancestral hapotype *k* carried by *j*-th animal; *σ*_*h*_^2^ is the haplotypic variance; and the covariance between ancestral haplotype effects is assumed to be zero. For this analysis, the polygenic effects (**u**) distributed as *N*(0, **A***σ*_*u*_^2^) and the residual effects (**e’**_i_) distributed as *N*(0, **I***σ*_*e* '_^2^) were derived in a similar manner as they were for the SNP-based GWAS. Using the GLASCOW software, a test statistics *T* based on the score tests was calculated at every tested position as follows;

$$ T=0.5\left({\mathbf{y}}_{\mathbf{adj}}-{\mathbf{1}}_{\mathbf{n}}\mu -\mathbf{u}\right)\mathbf{\hbox{'}}{\mathbf{H}}_i{\mathbf{H}}_i\mathbf{\hbox{'}}\left({\mathbf{y}}_{\mathbf{adj}}-{\mathbf{1}}_{\mathbf{n}}\mu -\mathbf{u}\right) $$

The *T* score can be interpreted as a sum over the ancestral haplotypes groups of the sum of the squared difference between observed (i.e., **y**_**adj**_) and expected (i.e., **1**_**n**_*μ* + **u**) values of the phenotypes for each group. The significance of the *T* scores can be obtained using the distribution of the *T* score under the null hypothesis (*σ*_*h*_^2^ = 0) that could be approximated using a gamma distribution. Parameters of the gamma distribution were estimated for each tested position.

### QTL detection

For both methods, the Bonferroni correction was applied to determine the 5 % genome-wide significance thresholds (*P* = 1.31 × 10^−6^). The extent of LD in this population was about 1.0 Mb, and the genome-wide significance thresholds defined by the Bonferroni correction was too conservative. Therefore, the genome-wide suggestive threshold was also defined as; *P* = 5.0 × 10^−5^. SNP maps were updated according to the SNPchiMp v.2 database [19] and the Sscrofa 10.2 assembly. The positional candidate genes within the range of 1 Mb bin size of the significant region were scanned using the NCBI2R R-package [20]. *r*^*2*^ values between the SNPs of the significant region were calculated, and the haplotype block pattern was visualized using Haploview software [21].

### Simulations

We used real genotype data and pedigree information in simulation analysis. The base phenotypes under the null hypothesis were simulated using a polygenic model with pedigree information [22]. The additive genetic value of *j*-th animal (*u*_*j*_) was generated based on data from the parent. If both parents were unknown (i.e., the animal is a founder), then *u*_*j*_ was derived as follows: *u*_*j*_ ~ *N*(0, *σ*_*u*_^2^). If both parents were known, *u*_*j*_ was derived as follows: *u*_*j*_ ~ *N*(0.5(*u*_*sj*_ + *u*_*Dj*_), 0.5(1 − 0.5(*F*_*Sj*_ + *F*_*Dj*_)*σ*_*u*_^2^)), where *u*_*Sj*_ and *u*_*Dj*_ are the additive genetic values of sire and dam of the *j*-th animal, respectively, and *F*_*Sj*_ and *F*_*Dj*_ are the inbreeding coefficient for the sire and dam of the *j*-th animal, respectively. The residual value of the *j*-th animal (*e*_*j*_) was derived from *e*_*j*_ ~ *N*(0, *σ*_*e*_^2^). The heritability of the base phenotype was set at 0.30, and phenotypic variance was assumed to be 1. The base phenotype was under the null hypothesis of no phenotype-SNP correlation (i.e., there was no significant effect for GWAS) (Additional file 4: Figure S3), and was used for simulation analysis.

Some missing genotypes were imputed by DualPHASE [12], and were used to generate QTL effects in 100 replicates. In each replicate, we simulated QTL effects under two factors: different QTL MAF categories and QTL heritabilities. Two QTL MAF categories were defined as follows: a low MAF group (0.01≤ MAF ≤0.10) and a high MAF group (0.10< MAF ≤0.5). Six set values of QTL heritability (0.01, 0.03, 0.05, 0.07, 0.10, and 0.15) were used. We carried out simulations in various combinations of QTL MAF categories (two analyses) and QTL heritabilities (six analyses). In each replicate, one SNP in the SNP array was randomly selected as the QTL and assigned a QTL effect derived by: $\pm \sqrt{\frac{\sigma_{QTL}^2}{2p\left(1-p\right)}}$; where *σ*_*QTL*_^2^ is the QTL variance and *p* is the allele frequency of the QTL across all animals. The signs of QTL effects were randomly selected, and the QTL variance was the same as the setting value of QTL heritability, because the phenotypic variance was assumed to be 1. The QTL effect was then added to the base phenotypic value. An error value was also added to generate a new phenotypic value with a setting value of heritability (0.30). The SNP selected as the QTL was then masked. In each replicate, SNP-based GWAS and haplotype-based GWAS were performed, and the power to achieve 5 % genome-wide significance (*P* = 1.31 × 10^−6^) was calculated as the proportion of replicates with a significant SNP within ± 0.5 Mb of the selected QTL. In addition, we also evaluated which method is practical for finer mapping in this population. The power to achieve 5 % genome-wide significance was calculated in three different regions, which ranged from ± 0.5 Mb, ± 0.5–1.0 Mb, and ± 1.0–2.0 Mb, apart from the selected QTL. In this scenario, the QTL heritability was set to 0.10.

### Phenotypes

Table 3 summarizes the data of the 52 traits of interest in the present study. Pigs were weighed at birth and at 21 day of age. From this group, pigs (barrows and gilts) that qualified for selection as candidates for the study and for full-sib tests were raised until they attained a body weight of 105 kg. The DG was calculated between 30 and 105 kg of body weight. Pigs were slaughtered at a live weight of approximately 105 kg. The day before slaughter, body weight, size, and length, including the circumference of the chest (CC) and cannon bone (CCB), the height at the withers (HEIGHT), and the chest depth (CD) and width (CW) of all animals were measured and recorded. Body composition traits comprised ultrasonically measured LEA and BF at half-body length. Structural soundness traits, such as those of the front and rear leg pasterns, were scored at body weights of 30 and 105 kg, based on a scale of 1 (straight) to 5 (soft). Scoring criteria and descriptions are presented in Additional file 5: Figure S4.

**Table 3:** Summary statistics of the study subjects

Trait	Abbreviation	N^a^	Mean	SD^b^	Minimum	Maximum
*Growth*						
Average daily gain from 30 to 105 kg body wt, g/d	DG	779	1094.00	112.80	750.6	1440.0
Ultrasound loin muscle area, cm^2^	LEA	776	34.64	3.27	26.0	50.2
Ultrasound backfat thickness, cm	BF	776	3.17	0.56	1.8	5.0
*Body measurements*						
Height at withers, cm	HEIGHT	779	60.97	1.98	54.4	67.4
Length, cm	LENGTH	779	106.00	3.38	98.0	116.0
Front width, cm	FW	779	34.72	1.49	30.0	42.5
Chest width, cm	CW	779	29.88	1.51	19.0	39.2
Rear width, cm	RW	779	32.29	1.21	28.6	38.8
Chest depth, cm	CD	779	35.77	1.28	24.9	39.8
Circumference of chest, cm	CC	779	111.40	3.01	99.0	120.0
Circumference of cannon bone at front (at 105 kg), cm	CCB at F105	779	18.17	0.67	16.0	20.6
Circumference of cannon bone at rear (at 105 kg), cm	CCB at R105	779	18.91	0.62	17.0	22.4
Circumference of cannon bone at front (at 30 kg), cm	CCB at F30	681	13.70	0.64	12.0	16.2
Circumference of cannon bone at rear (at 30 kg), cm	CCB at R30	681	14.05	0.64	12.4	16.5
Front leg score at 30 kg	SCORE at F30	718	2.70	0.54	1	5
Rear leg score at 30 kg	SCORE at R30	718	2.27	0.57	1	5
Front leg score at 105 kg	SCORE at F105	720	3.08	0.56	1	5
Rear leg score at 105 kg	SCORE at R105	720	2.73	0.57	1	5
*Carcass measurements*						
Carcass wt, kg	CWT	302	71.76	2.53	65.1	82.0
Carcass yield, %	CY	286	68.98	1.46	62.4	75.4
Carcass length (1st cervical - pubic), cm	CL	302	87.64	2.19	81.5	93.0
Carcass length I (1st thoracic - pubic), cm	CL1	302	72.34	1.95	67.0	77.6
Carcass length II (1st thoracic - last lumbus), cm	CL2	302	62.76	2.09	57.5	68.3
Carcass length III (Longissimus muscle length), cm	CL3	302	52.54	2.00	47.5	58.3
Carcass thickness, cm	CT	302	34.99	1.10	32.0	39.0
*Vertebrae number*						
Thoracic	TVN	302	14.71	0.67	13	16
Lumbar	LVN	302	6.03	0.50	5	8
Total	-	302	20.74	0.59	20	22
*Subcutaneous fat thickness*						
Shoulder, cm	SSFT	302	4.00	0.58	2.4	5.8
Back, cm	BSFT	302	2.59	0.45	1.6	4.0
Loin, cm	LSFT	302	3.30	0.46	2.2	6.7
*Longissimus muscle area*						
at 4–5 rib, cm^2^	LEA at 45r	301	17.85	3.18	9.0	31.2
at the middle, cm^2^	LEA at HBL	302	36.43	4.49	26.5	50.1
*Meat quality*						
Moisture content, %	MOS	302	72.72	1.16	68.8	75.3
Intramuscular fat, %	IMF	302	5.04	1.62	1.5	9.8
Protein content, %	PROT	302	21.34	0.66	19.5	23.4
Cooking loss, %	COOK	301	24.28	2.63	16.0	32.5
Centrifugal water-holding capacity, %	WHC	301	78.34	3.79	68.2	87.3
Shear force value, kg/cm^2^	SF	301	2.62	0.65	1.4	4.9
pH	pH	301	5.64	0.23	4.9	6.3
Lightness of longissimus muscle (L*)	M-L*	300	51.63	3.44	43.6	65.3
Redness of longissimus muscle (a*)	M-a*	300	3.55	1.18	0.7	6.9
Yellowness of longissimus muscle (b*)	M-b*	300	6.56	1.38	1.0	11.2
Lightness of subcutaneous fat (L*)	F-L*	301	78.34	2.35	71.6	90.1
Redness of subcutaneous fat (a*)	F-a*	301	3.50	0.00	1.3	0.6
Yellowness of subcutaneous fat (b*)	F-b*	301	7.84	1.10	5.3	10.8
*Fat area of carcass cross section at 4–5 rib*						
Subcutaneous fat area, cm^2^	SFA at 45r	280	81.95	15.70	47.5	131.7
Intermuscular fat area, cm^2^	IFA at 45r	280	76.81	16.27	33.2	124.8
All fat area, cm^2^	ALLFA at 45r	280	158.80	27.00	84.8	235.0
*Fat area of carcass cross section at the middle*						
Subcutaneous fat area, cm^2^	SFA at HBL	280	64.78	14.41	24.1	116.8
Intermuscular fat area, cm^2^	IFA at HBL	280	55.79	13.49	23.4	101.9
All fat area, cm^2^	ALLFA at HBL	280	120.60	25.54	57.6	218.8

^a^ *N* number records

^b^ *SD* standard deviation

Carcasses were scalded and dehaired, and chilled overnight. Carcass measurements were then recorded as follows: weight (CWT); lengths I (CL1), II (CL2), and III (CL3); thickness (CT); and vertebrae number. CL1, CL2, and CL3 denote the lengths from the first cervical vertebra to the pubic bone; from the first rib to the pubic bone; and from the first rib to last lumbar vertebra, respectively. Depth of backfat over the midline was measured with a ruler at the first rib (shoulder: SSFT), at the thinnest depth over the ribs (back: BSFT), and at the first lumbar vertebra (loin: LSFT). One side of the carcass was split between the fourth and fifth ribs (at 45r), and at half-body length (at HBL). The longissimus muscle was traced on acetate paper and the area was determined using computerized morphometric planimetry. Cross sections of each carcass were then recorded photographically. The fat area ratios for subcutaneous fat (SFA), intermuscular fat (IFA), and all fat area (ALLFA) ratios were measured at 45r and at HBL, using the ImageJ ver 1.48 software [23]. The classification descriptions are presented in Additional file 6: Figure S5.

The weight of each wholesale cut of the remaining side of the carcass was then recorded. Moisture (MOS), IMF, protein (PROT), cooking loss (COOK), water holding capacity (WHC), and shear force value (SF) were measured as described by Okumura et al. [24]. The MOS content was determined by drying approximately 2 g of a minced sample for 24 h at 105 °C. Using samples that had already been analyzed for MOS content, IMF content was determined by Soxhlet extraction of the dried samples with diethyl ether for 16 h. The PROT content was determined by the Kjeldahl method, using a nitrogen distillation titration device (2400 Kjeltec Auto Sampler System; FOSS, Hillerod, Denmark). The COOK was determined by placing approximately 50 g of a cube-shaped sample of meat into a sealed plastic bag, which was then placed in a water bath for 1 h at 70 °C. For SF determination, at least four pieces that had already been analyzed for COOK were cut (vertical cross section 1 cm × 1 cm) parallel to the long axis of the muscle fibers. Each piece was sheared with a Warner-Bratzler shear device attached to an Instron Universal Testing Machine (Model 5542; Instron Japan Co. Ltd., Kanagawa, Japan) with a cross head speed of 200 mm/min. The WHC was determined by centrifugation of approximately 500 mg of a cube-shaped sample of meat at 2380 *g* for 30 min at 2 °C. The pH of the longissimus muscle was measured using a pH meter (D-51, HORIBA Ltd., Kyoto, Japan) equipped with a needle-type electrode (6252-10D, HORIBA Ltd.). The appearance of meat and fat (lightness L*, redness a*, and yellowness b*) were assessed immediately after cutting, using a Minolta spectrophotometer CM-2002 (Minolta Camera Co. Ltd., Osaka, Japan). All measurements were performed in duplicate, and means and standard errors were calculated.

## Results

### Comparison of the two methods in simulation and real data analysis

**Fig. 1:**
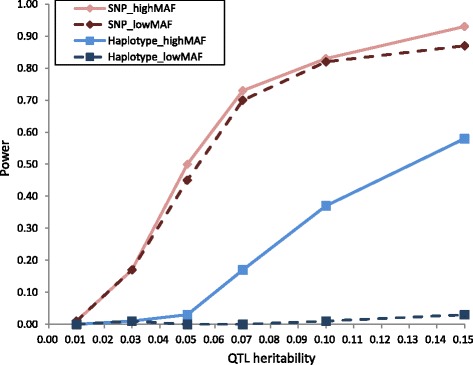
Power to achieve 5 % genome-wide significance in simulation analysis. The x-axis indicates QTL heritability and the y-axis represents the power to detect QTL. Results of varying minor allele frequency (MAF) categories (low and high) and models (SNP-based and haplotype-based genome-wide association studies) are shown

The powers to detect QTL by SNP-based GWAS and haplotype-based GWAS in simulation analysis are presented in Fig. 1. With regard to the impact of QTL MAF on QTL detection, the difference in power between SNP-based GWAS and haplotype-based GWAS was evident. For SNP-based GWAS, the similar trend of the results was observed in the power to detect QTL with high and low MAFs. For haplotype-based GWAS, the power to detect QTL with high MAF was greater, as the QTL heritability increased to more than 0.05. However, the power to detect QTL with low MAF was quite low at all QTL heritabilities (the maximum value of power was 0.03). The power of SNP-based GWAS was greater than that of haplotype-based GWAS under all simulation conditions. For SNP-based GWAS, as the QTL heritability increased, the power to detect QTL also increased and was almost constant at higher QTL heritabilities (more than 0.10). In addition, the power to detect QTL with heritability 0.05 was 0.50 in a high-MAF scenario and 0.45 in a low-MAF scenario. Thus, QTL with smaller heritabilities and both MAFs could be detected by SNP-based GWAS. For haplotype-based GWAS, the power to detect QTL with heritability less than 0.05 was very low (less than 0.03) in a high MAF scenario, but increased as the QTL heritability increased to more than 0.05.

**Fig. 2:**
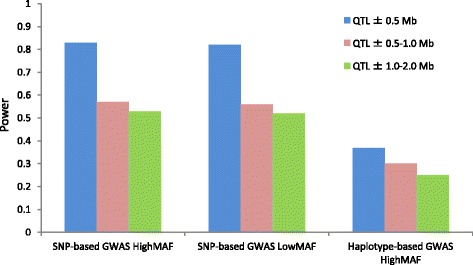
Power to achieve 5 % genome-wide significance within different ranges around selected QTL in simulation analysis. The y-axis represents the power to detect QTL. The results of the SNP-based genome-wide association study (GWAS) in high and low minor allele frequency (MAF) scenarios and those of haplotype-based GWAS in a high MAF scenario are shown. Three different ranges around the selected QTL were evaluated. QTL ± 0.5 Mb: The region ranged from ±0.5 Mb apart from the selected QTL. QTL ± 0.5–1.0 Mb: The region ranged from ±0.5 to 1.0 Mb apart from the selected QTL. QTL ± 1.0–2.0 Mb: The region ranged from ±1.0 to 2.0 Mb apart from the selected QTL

For the practicability of finer mapping by SNP- and haplotype-based GWAS, the power among three different region apart from the selected QTL were calculated by SNP- and haplotype-based GWAS, and are shown in Fig. 2. The power of haplotype-based GWAS in a low-MAF scenario are not shown, because of very low power (see Fig. 1). For SNP-based GWAS, the similar trend of the results was observed in the power to detect QTL with high and low MAFs. The power decreased from the region (QTL ± 0.5 Mb) to the region (QTL ± 0.5–1.0 Mb) was a greater extent than it did from the region (QTL ± 0.5–1.0 Mb) to the region (QTL ± 1.0–2.0 Mb), and the decreased power was 0.26 and 0.04, respectively On the other hand, the power of the haplotype-based GWAS showed a constant decrease, and the decreased power of the region of interest was 0.07 and 0.05, respectively.

**Fig. 3:**
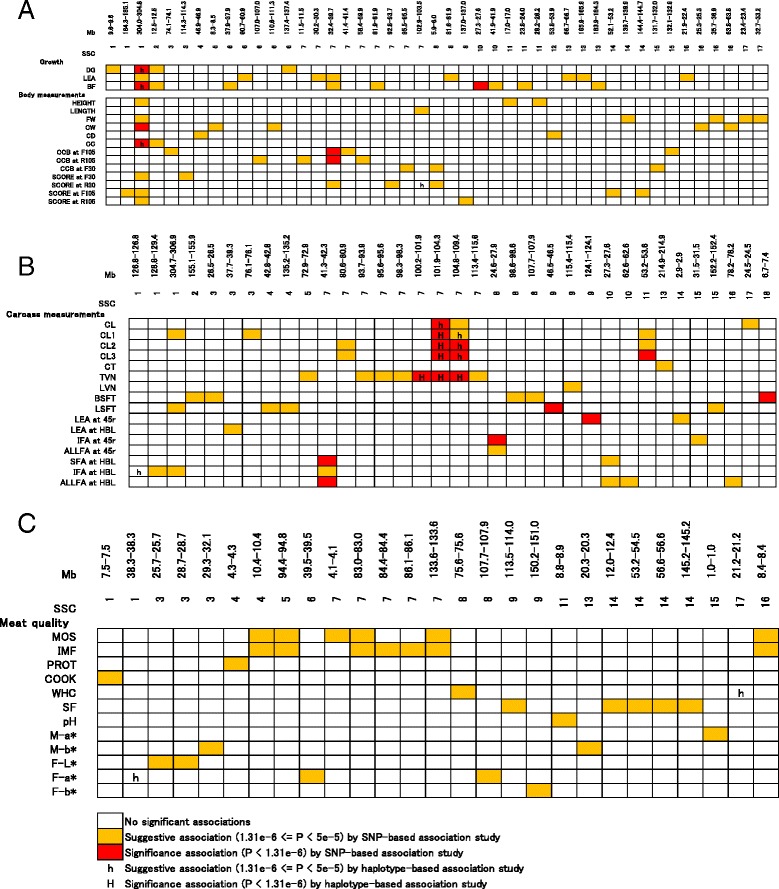
Trait associations across genomic regions analyzed by SNP-based and haplotype-based genome-wide association studies (GWAS). *Each row* represents a trait, and *each column*, a genomic region containing SNPs that are genome-wide suggestively or significantly associated with a trait. Only traits with at least one associated SNP and SNPs associated with at least one trait are shown. Each summary shows the results of growth traits (**a**) carcass traits (**b**) and meat quality traits (**c**). SSC, *Sus scrofa* chromosome; DG, Average daily gain; LEA, Ultrasound loin muscle area; BF, Ultrasound backfat thickness; HEIGHT, Height at withers; FW, Front width; CW, Chest width; CD, Chest depth; CC, Circumference of chest; CCB at F(/R) 30(/105), Circumference of cannon bone at front (/Rear) (at 30 kg/105 kg); SCORE at F(/R) 30(/105), Front (/Rear) leg score at 30 kg (/105 kg); CL, Carcass length; CL1, Carcass length I; CL2, Carcass length II; CL3, Carcass length III; CT, Carcass thickness; TVN, Thoracic vertebrae number; LVN, Lumbar vertebrae number; BSFT, Subcutaneous fat thickness (Back); LSFT, Subcutaneous fat thickness (Loin); 45r, carcass cross section at fourth–fifth rib; HBL, carcass cross section at half-body length; LEA at 45r, Longissimus muscle area at 45r; LEA at HBL, Longissimus muscle area at HBL; IFA at 45r, Intermuscular fat area at 45r; ALLFA at 45r, All fat area of 45r; SFA at HBL, Subcutaneous fat area at HBL; IFA at HBL, Intermuscular fat area at HBL; ALLFA at HBL, All fat area at HBL; MOS, Moisture; IMF, Intramuscular fat; PROT, Protein; COOK, Cooking loss; WHC, Centrifugal water-holding capacity; SF, Shear force value; M-a*, Redness of longissimus muscle; M-b*, Yellowness of longissimus muscle; F-L*, Lightness of subcutaneous fat; F-a*, Redness of subcutaneous fat; F-b*, Yellowness of subcutaneous fat

In real data analysis, SNP-based GWAS and haplotype-based GWAS were performed for 52 traits related to growth, carcass, and meat quality. We summarized the genome-wide significant and suggestive SNP regions for these traits in Fig. 3 and Additional file 7: Table S2. For SNP-based GWAS, 23 genome-wide significant SNP regions were detected in 17 traits, and 120 genome-wide suggestive SNP regions were detected in 27 traits. For haplotype-based GWAS, 6 genome-wide significant SNP regions were detected in four traits, and 11 genome-wide suggestive SNP regions were detected in eight traits. All genome-wide significant SNP regions detected by haplotype-based GWAS were located in regions that were also detected by SNP-based GWAS. Most of the genome-wide suggestive SNP regions detected by haplotype-based GWAS were located in regions that were also detected by SNP-based GWAS. However, four of these regions were detected by haplotype-based GWAS only.

### QTL detection

**Fig. 4:**
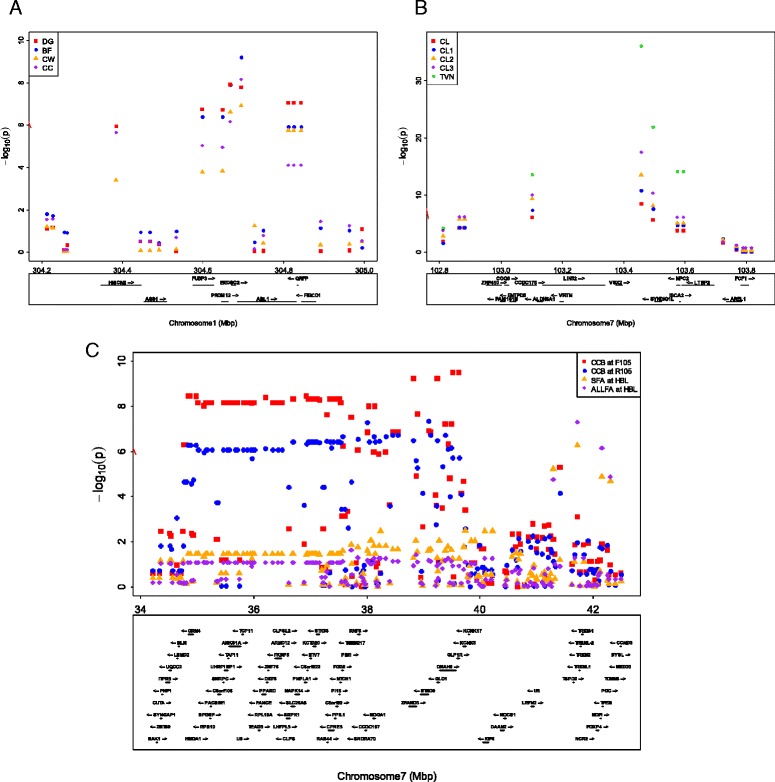
Regional plots of several loci associated with 13 traits. The x-axis indicates the Mb, and the y-axis indicates -log_10_ (*p*-value). Gene loci and their strands were annotated based on Sscrofa10.2 assembly from the Ensemble database (http://asia.ensembl.org/Sus_scrofa/Info/Index?db=core). *Dashed line* indicates the threshold of the Bonferroni 5 % genome-wide significance level. **a** Plots in chromosome 1 (304.2–305.0 Mb) for average daily gain (DG), backfat thickness (BF), chest width (CW), and circumference of chest (CC). **b** Plots in chromosome 7 (102.8–103.9 Mb) for Carcass length (CL), Carcass length I (CL1), Carcass length II (CL2), Carcass length III (CL3), and Thoracic vertebrae number (TVN). **c** Plots in chromosome 7 (34.2–42.5 Mb) for circumference of cannon bone at front (CCB at F105), circumference of cannon bone at rear (CCB at R105), Subcutaneous fat area of carcass cross section at half-body length (SFA at HBL), and All fat area of carcass cross section at half-body length (ALLFA at HBL)

The SNPs showing genome-wide significant association with growth, body measurements, carcass measurements, and fat area in SNP-based GWAS and haplotype-based GWAS are presented in Table 4. In addition, three regional plots associated with multiple traits are shown in Fig. 4. Significant association between rs81352956 on *Sus scrofa* chromosome (SSC) 1 at 304 Mb and DG (*P* = 1.22 × 10^−8^) was evident in both SNP-based and with haplotype-based analyses (Table 4, Fig. 4a). Each copy of effect allele A at rs81352956 was associated with a decrease in DG of 34.75 g/d. Moreover, significant associations between rs81352969 on SSC1 at 304 Mb and BF (*P* = 6.30 × 10^−10^) and CC (*P* = 6.82 × 10^−9^), respectively, were observed in both SNP-based and haplotype-based analyses (Table 4). However, a significant association between rs81352969 on SSC1 at 304 Mb and CW (*P* = 1.17 × 10^−7^) was only detected with SNP-based GWAS. Each copy of effect allele A at rs81352969 was associated with increases in BF of 0.18 cm; CW of 0.45 cm; and CC of 0.87 cm. Both SNPs, rs81352969 and rs81352956 on SSC1 at 304 Mb, were located within close proximity (a distance of 27 kb) of each other.

**Table 4:** Top genome-wide significant SNPs associated with growth, body measurements, carcass measurements and fat area

		Most significant SNP								Haplotype-based	Nearby genes^g^
Trait^a^		SSC^b^	Position^c^ (bp)	refSNP variation ID	EA^d^	EAF^d^	β (SE)	Proportion^e^	*P*-value	*P*-value^f^	
*Growth*											
	DG	1	304,667,314	rs81352956	A	0.454	−34.75 (6.035)	0.06	1.22 × 10^−8^	6.71 × 10^−6^ h	*PRDM12_EXOSC2*
	BF	1	304,694,455	rs81352969	A	0.495	0.18 (0.029)	0.07	6.30 × 10^−10^	2.26 × 10^−5^ h	***ABL1***
		10	27,636,391	rs81422289	G	0.227	0.17 (0.034)	0.04	7.04 × 10^−7^	3.63 × 10^−2^	***KIF14***
*Body measurements*											
	CW	1	304,694,455	rs81352969	A	0.495	0.45 (0.084)	0.05	1.17 × 10^−7^	1.45 × 10^−4^	***ABL1***
	CC	1	304,694,455	rs81352969	A	0.495	0.87 (0.148)	0.06	6.82 × 10^−9^	3.36 × 10^−5^ h	***ABL1***
	CCB at F105	7	39,512,713	rs80892802	G	0.171	0.29 (0.045)	0.07	3.14 × 10^−10^	1.75 × 10^−3^	***DNAH8***
	CCB at R105	7	39,089,506	rs196955082	G	0.249	0.21 (0.039)	0.05	4.38 × 10^−8^	1.19 × 10^−3^	***BTBD9***
*Carcass measurements*											
	CL	7	103,457,401	VRTN	C	0.368	1.13 (0.187)	0.14	3.64 × 10^−9^	3.36 × 10^−6^ h	***VRTN***
	CL1	7	103,457,401	VRTN	C	0.368	1.13 (0.162)	0.18	1.56 × 10^−11^	4.87 × 10^−8^ H	***VRTN***
	CL2	7	103,457,401	VRTN	C	0.368	1.37 (0.172)	0.22	2.96 × 10^−14^	2.22 × 10^−9^ H	***VRTN***
		7	107,279,922	rs80977788	G	0.267	−0.97 (0.196)	0.09	1.20 × 10^−6^	1.25 × 10^−5^ h	***NRXN3***
	CL3	7	103,457,401	VRTN	C	0.368	1.48 (0.159)	0.27	3.10 × 10^−18^	1.94 × 10^−11^ H	***VRTN***
		7	106,308,596	rs80813652	G	0.311	1.02 (0.177)	0.12	2.14 × 10^−8^	1.55 × 10^−5^ h	***LOC102158165***
		11	53,204,914	rs330963199	G	0.253	−1.00 (0.191)	0.10	3.32 × 10^−7^	1.31 × 10^−2^	***LOC102167198***
	TVN	7	101,863,838	rs80966250	G	0.349	−0.35 (0.059)	0.13	9.93 × 10^−9^	4.44 × 10^−16^ H	*LOC102164420_LOC102164550*
		7	103,457,401	VRTN	C	0.368	0.67 (0.046)	0.50	9.42 × 10^−37^	4.44 × 10^−16^ H	***VRTN***
		7	106,308,596	rs80813652	G	0.311	0.44 (0.056)	0.20	5.17 × 10^−14^	2.67 × 10^−9^ H	***LOC102158165***
	BSFT	18	7,107,781	rs81345146	A	0.361	−0.19 (0.037)	0.10	4.37 × 10^−7^	7.80 × 10^−4^	***ZYX***
	LSFT	9	46,466,374	rs81257576	A	0.017	0.65 (0.129)	0.07	7.36 × 10^−7^	4.41 × 10^−1^	*ZBTB16_NNMT*
	LEA at 45r	9	124,098,143	rs81415869	G	0.023	3.98 (0.746)	0.10	1.94 × 10^−7^	8.35 × 10^−1^	*LOC102162561_LOC100524389*
*Fat area of carcass cross section at 4–5 rib*											
	IFAat 45r	8	25,653,506	rs81398418	A	0.279	7.15 (1.380)	0.09	4.18 × 10^−7^	1.14 × 10^−4^	*LOC100624133_LOC102162593*
*Fat area of carcass cross section at the middle*											
	SFA at HBL	7	41,720,015	rs80928067	G	0.030	−16.52 (3.219)	0.11	5.35 × 10^−7^	5.11 × 10^−1^	***LOC100737927***
	ALLFA at HBL	7	41,720,015	rs80928067	G	0.030	−30.43 (5.433)	0.13	5.09 × 10^−8^	5.41 × 10^−1^	***LOC100737927***

^a^Abbreviations of trait are shown in Table 2

^b^ *SSC Sus Scrofa* chromosome

^c^Position for genome build 10.2

^d^ *EA* effect allele, *EAF* effect allele frequency

^e^The proportion of phenotypic variance explained by the SNP effects

^f^The results of haplotype-based association study are indicated by h = suggestive, H = significant difference

^g^Nearby genes are bolded if SNP is within the reference gene

Significant associations of one SNP derived from the vertnin (*VRTN*) gene on SSC7 at 103 Mb with CL (*P* = 3.64 × 10^−9^), CL1 (*P* = 1.56 × 10^−11^), CL2 (*P* = 2.96 × 10^−14^), CL3 (*P* = 3.10 × 10^−18^), and thoracic vertebrae number (TVN, *P* = 9.42 × 10^−37^) were observed in both SNP-based and haplotype-based analyses (Table 4, Fig. 4b). Each copy of the C allele was associated with an estimated increase in the TVN of 0.67 and the proportion of variance in TVN explained by an SNP was about 50 %.

Two regions on SSC7, at 35–39 Mb and 41–42 Mb, respectively, were associated with multiple traits. The region at 35–39 Mb was associated with CCB both at the front and rear at 105 kg body weight (*P* = 3.14 × 10^−10^ and *P* = 4.38 × 10^−8^, respectively; Table 4, Fig. 4c). Each copy of the G allele was associated with an estimated increase in CCB of 0.29 and 0.21 cm, respectively. This region was related to the formation of a long-range LD block from 35.0 to 37.5 Mb (see Additional file 8: Figure S6). Significant associations between rs80928067 on SSC7 at 41–42 Mb with SFA at HBL and ALLFA at HBL (*P* = 5.35 × 10^−7^ and *P* = 5.09 × 10^−8^, respectively) were detected with SNP-based GWAS only (Table 4). Each copy of effect allele G (with MAF 0.030) at rs80928067 was associated with a decrease in SFA at HBL of 16.52 cm^2^ and in ALLFA at HBL of 30.43 cm^2^.

Significant SNPs were detected within six regions with SNP-based GWAS only. Significant association between rs81398418 on SSC8 at 25 Mb and IFA at 45r (*P* = 4.18 × 10^−7^) was detected (Table 4), which accounts for 9 % of the phenotypic variance. Each copy of the A allele was associated with an estimated increase in IFA at 45r of 7.15 cm^2^. Significant association between rs81257576 on SSC9 at 46 Mb and LSFT (*P* = 7.36 × 10^−7^) was detected. Each copy of the A allele (with MAF 0.017) was associated with an estimated increase in LSFT of 0.65 cm. Significant association between rs81415869 on SSC9 at 124 Mb and LEA at 45r (*P* = 1.94 × 10^−7^) was detected and accounts for 10 % of the phenotypic variance. Each copy of the G allele (with MAF 0.023) was associated with an estimated increase in LEA at 45r of 3.98 cm^2^. Significant association between rs81422289 on SSC10 at 27 Mb and BF (*P* = 7.04 × 10^−7^) was detected. Each copy of the G allele was associated with an estimated increase in BF of 0.17 cm. Significant association between rs330963199 on SSC11 at 53 Mb and CL3 as longissimus muscle length (*P* = 3.32 × 10^−7^) was detected and accounts for 10 % of the phenotypic variance. Each copy of the G allele was associated with an estimated decrease in longissimus muscle length of 1.00 cm. Significant association between rs81345146 on SSC18 at 7 Mb and BSFT (*P* = 4.37 × 10^−7^) was detected and accounts for 10 % of the phenotypic variance. Each copy of the A allele was associated with an estimated decrease in BSFT of 0.19 cm.

No associations were detected in meat quality traits at the genome-wide significance threshold; however, 27 regions were suggested to be associated with meat quality traits (Fig. 3 and Additional file 7: Table S2). We discuss further the regions associated with IMF in greater detail, because this population was generated for the purpose of improving IMF content. Average IMF content was 5.04 % (SD = 1.62) with a minimum of 1.5 % and a maximum of 9.8 % in this study. The mean breeding value of IMF content in each generation was as follows: generation 1 (G1) = −0.000; G2 = −0.003; G3 = 0.075; G4 = −0.064; G5 = 0.230; G6 = 0.581; and G7 = 1.107. Suggestive association between rs80793147 on SSC4 at 10 Mb and IMF content (*P* = 1.09 × 10^−5^) was detected with SNP-based GWAS only. Each copy of the A allele was associated with an estimated decrease in IMF content of 0.59 %. Suggestive association between rs336391107 on SSC7 at 83 Mb and IMF content (*P* = 5.78 × 10^−6^) was detected with SNP-based GWAS only. Each copy of the A allele was associated with an estimated decrease in IMF content of 0.58 % (Additional file 7: Table S2). Each of the two SNPs associated with IMF accounted for 7 % of the phenotypic variance.

## Discussion

### Comparison of the two methods in simulation and real data analysis

In the present study, a comparison of the power to detect QTL between SNP-based GWAS and haplotype-based GWAS was made using simulation and real data. Several significant regions were detected by both methods with real data. For example, the significant region on SSC7 was detected for TVN, and the variant in the *VRTN* gene was also located in this region. The *VRTN* gene is the candidate gene that affects TVN [25]; thus, TVN was reliably affected by the detected region. The range of the significant region detected by haplotype-based GWAS was larger than that detected by SNP-based GWAS (Additional file 9: Figure S7A). This trend was similar to that observed in the simulation study. In the simulation study, we also evaluated the power among three regions, which ranged from ± 0.5 Mb, ± 0.5–1.0 Mb, and ± 1.0–2.0 Mb, apart from the selected QTL. As the region of interest grew further apart from the selected QTL, the power of the SNP-based GWAS decreased. In particular, the power decreased from the region of ± 0.5 Mb to the region of ± 0.5–1.0 Mb to a greater extent than it did from the region of ± 0.5–1.0 Mb to the region of ± 1.0–2.0 Mb. On the other hand, the power of the haplotype-based GWAS showed a constant decrease, as the region of interest grew further apart from the selected QTL. The most significant differences between SNP-based GWAS and haplotype-based GWAS are due to differences in the number of generations considered [8]. Recombination events are scored over a limited number of generations in the pedigree for haplotype-based GWAS, whereas SNP-based GWAS relies on a large number of historical recombination events in past generations. Recombination events with SNPs around the selected QTL can facilitate finer mapping in the QTL region. Previous studies have reported comparisons of both methods in half-sib and F_2_ intercross pig populations [9–11]. The present study used a multigenerational population, in which the chances of a recombination or segregation event occurring during meiosis would be greater than they would be in a half-sib or F_2_ intercross population. However, finer mapping is still not practical in this population. Therefore, SNP-based GWAS is recommended for fine mapping, provided the significant region can be detected by both methods.

In real data analysis, a larger number of significant regions was detected by SNP-based GWAS than by haplotype-based GWAS. For example, the significant region on SSC7 was detected for ALLFA at HBL by analysis of the SNP-based GWAS. However, the same region was not detected by haplotype-based GWAS (Additional file 9: Figure S7B). This trend was similar to that observed in the simulation study, and the power of SNP-based GWAS was higher than that of haplotype-based GWAS under all simulation conditions. Legarra et al. [10] reported that haplotype-based GWAS yielded lower power and higher rates of false positives in simulation, in comparison to SNP-based GWAS. This finding is consistent with the results of the present study as we also demonstrated the greater power of SNP-based GWAS in comparison to that of haplotype-based GWAS in a multigenerational population. Therefore, a novel region could be detected by SNP-based GWAS.

In the present study, several significant regions were detected in real data by haplotype-based GWAS only. For example, the genome-wide suggestive region on SSC17 was detected for WHC by analysis of the haplotype-based GWAS. However, the significant region was not detected by SNP-based GWAS (Additional file 9: Figure S7C). In the simulation study, a significant region detected by haplotype-based GWAS (but not by SNP-based GWAS) was observed in one replicate only under all simulation conditions (Additional file 10: Table S3). This suggested that only SNP-based GWAS or a combination of the two methods could reliably detect QTL, provided one SNP is assumed to be the QTL. In certain cases, linkage-based GWAS can detect rare variants by aggregate analysis as opposed to individual analysis [8]. In the simulations of the present study, only one SNP was assumed to be the QTL, and the phenotypic value was then generated. In that case, the power of the SNP-based GWAS was greater than that of haplotype-based GWAS in a low MAF scenario. Thus, several rare variants in the regions of interest might have caused the observed effects on the trait under investigation. Another possibility is the effect of the copy number variant (CNV), which could not be accounted for in the SNP array. Recent studies have reported the association between CNV and economically important traits in pigs [26, 27]. In real data analysis of haplotype-based GWAS of color-sidedness traits in a cattle population, Zhang et al. [18] reported that ancestral haplotypes presented high LD with CNVs that had been previously reported by Durkin et al. [28]; however, the same region was not significant following analysis of SNP-based GWAS. Thus, haplotype-based GWAS can capture variants associated with the CNV, and further study is needed to detect the variant(s) around the significant regions.

### QTL detection

In the population of the present study, four regions were associated with multiple traits. One region on SSC1 at 304 Mb that included rs81352956 was associated with DG, BF, CW, and CC. This region was in close proximity to the locus at which the far upstream element (FUSE) binding protein 3 (*FUBP3*) gene (which is involved in *c-Myc* regulation) is located. In humans, this locus is associated with height [29] and bone mineral density [30]. The candidate gene *FUBP3* may affect skeletal formation as it relates to both height and bone mineral density. A region on SSC7 at 103 Mb was associated with CL and TVN. Mikawa et al. [25] reported that the *VRTN* gene in this region is responsible for the QTL for the TVN in commercial breeds of pigs. Two of nine polymorphic sites, an insertion of the PRE1 element in intron 1 and an SNP in the promoter region, may have effected changes in expression of the *VRTN* gene at the embryonic stage. Fan et al. [31] reported complete LD between AB554652.1:g.19034A > C in the promoter region and g.20311_20312ins291 in intron 1 as the causal mutation. In the present study, we used the presence or absence of an insertion of the PRE1 element in intron 1 as a marker for simple diagnosis. An allele of *VRTN* affects the TVN, CL, and the length of the longissimus muscle. These findings are consistent with those of Nakano et al. [32] who reported that *VRTN* gene polymorphisms greatly contributed to the TVN and CL-related traits. Two regions on SSC7 at 35–39 Mb and 41–42 Mb had genome-wide significance with multiple traits. In the region at 35–39 Mb, an LD block from 35.0 to 37.5 Mb was observed, which is consistent with the report of Guo et al. [33]. A long-range LD block in this region and the positions of several genes could interfere with the detection of polymorphism(s) responsible for CCB. The region on SSC7 at 35 Mb was in close proximity to the locus at which the high mobility group AT-hook 1 (*HMGA1*) gene (which is involved in many cellular processes, including cell growth and differentiation) is located. A QTL for the CCB in Duroc pigs has been reported in this region [34]. In addition, a QTL for limb bone lengths in a Duroc crossed population has been reported by Guo et al. [35] in the same region. In humans, *HMGA2* is one of the strong biological candidates for height [36]. Therefore, *HMGA1* is possibly also a strong candidate gene that might improve leg strength in Duroc sires that are used in terminal crosses. The region at 41–42 Mb including rs80928067 was associated with SFA at HBL and was in close proximity to the locus at which the triggering receptor expressed on myeloid cells 2 (*TREM2*) gene (which is involved in osteoclast development and the anti-inflammatory response) is located. In humans, this gene is identified as one with transcripts exhibiting differential patterns of expression in abdominal subcutaneous fat between obese and normal pregnant women [37]. In the mouse, *TREM2* is required for adipocyte differentiation and promotes adipogenesis by upregulating adipogenic regulators and inhibiting the Wnt10b/β-catenin signaling pathway [38]. Therefore, we suggest that the *TREM2* gene may be a candidate for the quantitative trait of fat deposition.

In the present study, six regions had genome-wide significance, and three of those six regions contained potential candidate genes. The region on SSC9 at 124 Mb including rs81415869 was associated with LEA at 45r and was in close proximity to the locus at which the thiamine pyrophosphokinase (*TPK1*) gene (which is involved in thiamine metabolism) is located. TPK1 is a cellular enzyme that catalyzes the transfer of the pyrophosphate group from ATP to thiamine, to form thiamine pyrophosphate (TPP). TPP is an active cofactor for enzymes involved in glycolysis and energy generation. In humans, QTL for birth weight have been mapped on chromosome 7, using linkage analysis [39] and *TPK1* has been tested as a candidate gene [40]. With respect to birth weight, phenotype was markedly influenced by skeletal muscle mass, and *TPK1* may be a candidate gene for the quantitative trait LEA at 45r. The region on SSC10 at 27 Mb including rs81422289 was associated with BF and was in close proximity to the zinc finger protein 281 (*ZNF281*) gene, which was predicted to be a potential target of microRNA-33 (miR-33) [41]. In mice, miR-33 coordinates genes that regulate progression of the cell cycle, fatty acid and glucose metabolism, and cholesterol homeostasis [42]. Therefore, we suggest that *FUBP3* and *ZNF281* may be candidate genes for the quantitative traits DG and BF. The region on SSC18 at 7 Mb including rs81345146 was associated with BSFT and was in close proximity to the locus at which the zyxin (*ZYX*) gene (which has N-terminal proline-rich repeats and three copies of LIM (Lin-11, IsI-I, and Mec3) domains in its C-terminal half [43]) is located. Macalma et al. [43] suggested that *ZYX* is involved in a number of important signaling pathways that regulate cell differentiation, proliferation, and morphology. Proline-rich and LIM domains interacted with a number of proteins and specific protein partners, respectively. One of those partners, the Homeodomain-interacting protein kinase 2 (*HIPK2*) gene, identified as an essential regulator of white fat development, might have functions within the adipocyte cell compartment of the skin in the mouse [44]. Therefore, we suggest that *ZYX* may be a candidate gene for quantitative trait of fat deposition.

In the present study, we observed no genome-wide significant association with IMF. However, two regions with genome-wide suggestive association contained potential candidate genes. The region on SSC4 at 10 Mb including rs80793147 was associated with IMF content and was in close proximity to the locus at which the ArfGAP with SH3 domain, ankyrin repeat, and PH domain 1 (*ASAP1*) gene (also known as differentiation-enhancing factor; *DEF-1*) is located. DEF-1 is involved in the differentiation of fibroblasts and possibly that of other cell types. In vitro, this gene encoded a Src SH3 binding protein, increased expression of DEF-1, promoted adipogenesis in cultured fibroblast cell lines, and was detected in the adipose tissue of obese and diabetic mice. In addition, the ubiquitous expression pattern of DEF-1 implies that its function is not restricted to adipogenesis [45]. The region on SSC7 at 83 Mb including rs336391107 was associated with IMF content, and was in close proximity to the locus at which the methyltransferase like 3 (*METTL3*) gene (which is involved in messenger RNA modification of N^6^-methyladenosine (m^6^A) methylation) is located. Adenosine methylation in RNA is a reversible modification that is widespread throughout the transcriptome, and plays an important role in RNA biology, which influences a wide variety of biological pathways and physiological processes [46]. Fustin et al. [47] reported that inhibition of m^6^A RNA–methylation by *METTL3* suppression reduced RNA processing efficiency. In porcine adipocytes, overexpression of *METTL3* significantly reduced the intracellular triglyceride content, significantly increased glycerol content in the medium, and significantly downregulated relative mRNA expression of proliferator-activated receptor γ and fatty acid synthase genes [48]. *METTL3* also enhanced m^6^A levels and inhibited adipogenesis. Therefore, we suggest that *METTL3* and *DEF-1* may be candidate genes for the quantitative trait of IMF content.

As we expected that the *VRTN* is a responsible gene for TVN [25, 31], only one of the 14 known candidate genes, *VRTN*, was confirmed to have significant association with TVN as well as CL. We were unable to confirm any significant associations, even at a suggestive level, for the other 13 known candidate genes. These findings could be attributed to the fact that the MAFs of candidate genes were low (e.g., the MAF of *CYB5A*, *ALGA0113531*, *LEPR*, and *PIK3C3* were 0.072, 0.098, 0.025, and 0.032, respectively). The effects of candidate genes depend on the genetic background of the population. Further study is necessary to evaluate the effects of candidate genes in different genetic backgrounds. Nevertheless, we identified multiple novel regions related to growth, carcass, and meat quality traits, which were separate from the regions of known candidate genes. The annotation of these genomic regions exhibited several genes that function in the regulation of *c-Myc*, cell growth and differentiation, thiamine metabolism, and adipogenesis. These findings provide new insights for investigations into altered gene functions that result from modifications of RNA and/or micro-RNA, in addition to DNA polymorphisms. Our results also suggest that the detected region could be useful as a means of improving marker-assisted selection in pigs.

## Conclusions

SNP-based and haplotype-based GWAS were performed in a Duroc multigenerational population. A comparison was drawn between the power of SNP-based GWAS and that of haplotype-based GWAS, using simulation and real data; SNP-based GWAS demonstrated greater power than haplotype-based GWAS in the population under investigation. In real data analysis, larger numbers of significant regions were obtained and some regions had genome-wide significant association with multiple traits. In these significant regions, genes that serve specific functions were located. Among them, *FUBP3*, a gene implicated in *c-myc* regulation, is highlighted as novel candidate gene for skeletal formation associated with average daily gain, and *METTL3*, a gene implicated in messenger RNA modification, is novel candidate gene for fat deposition associated with IMF content. Further research could identify the causal genes involved in growth, carcass, and meat quality traits.

## Ethics (and consent to participate)

All procedures involving animals followed the Guidelines for the Care and Use of Laboratory Animals established by the National Livestock Breeding Center, and this research project was approved by the laboratory animals committee (23–9) on the National Livestock Breeding Center.

## Consent to publish

Not applicable.

## Availability of supporting data and materials

The datasets supporting the conclusions of this article are included within the article and its additional files.

## Additional files

Additional file 1: Figure S1. Boxplot of inbreeding coefficient in each generation. (PDF 167 kb) Additional file 2: Table S1. Genetic markers associated with production traits and related polymorphisms from a study of candidate genes. (XLSX 17 kb) Additional file 3: Figure S2. Average linkage disequilibrium coefficient (r^2^) values plotted against intermarker distance for all autosomal chromosomes. (PDF 407 kb) Additional file 4: Figure S3. Quantile-quantile plot of the *P*-value for SNP-based genome-wide association study in base phenotype. (PDF 279 kb) Additional file 5: Figure S4. Scoring criteria for feet and leg structure soundness traits. (PDF 320 kb) Additional file 6: Figure S5. Classification of fat accumulation of carcass cross sectional image. (PDF 206 kb) Additional file 7: Table S2. SNPs showing genome-wide significant and suggestive association with multiple measurements of growth, carcass, and meat quality traits. (XLSX 42 kb) Additional file 8: Figure S6. Linkage disequilibrium (LD) of the significant region on chromosome 7. (PDF 415 kb) Additional file 9: Figure S7. Comparison of the regional plots by SNP-based and haplotype-based genome-wide association studies (GWAS). (PDF 285 kb) Additional file 10: Table S3. Number of replicates for detection of significant SNPs in simulation study. (XLSX 10 kb)

## Abbreviations

ASAP1: ArfGAP with SH3 domain, ankyrin repeat, and PH domain 1
BLUP: best liner unbiased prediction
CNV: copy number variant
DEF-1: differentiation-enhancing factor
DG: average daily gain
FUBP3: far upstream element (FUSE) binding protein 3
GEMMA: genome-wide efficient mixed-model association
GWAS: genome-wide association study
HIPK2: homeodomain-interacting protein kinase 2
LD: linkage disequilibrium
LEPR: leptin receptor
LIM: Lin-11, IsI-I, and Mec3
MAF: minor allele frequency
METTL3: methyltransferase like 3
miR-33: microRNA-33
m^6^A: N^6^-methyladenosine
QTL: quantitative trait locus
SCD: stearoyl-CoA desaturase
SNP: single nucleotide polymorphism
SSC: *Sus Scrofa* chromosome
TPK1: thiamine pyrophosphokinase
TPP: thiamine pyrophosphate
TREM2: triggering receptor expressed on myeloid cells 2
VRTN: vertnin
ZNF281: zinc finger protein 281
ZYX: zyxin
