# Supplementary material for: SNP- and haplotype-based genome-wide association studies for growth, carcass, and meat quality traits in a Duroc multigenerational population
Source: BMC Genet. 2016 Apr 19;17:60. doi: 10.1186/s12863-016-0368-3 (PMC4837538; doi:10.1186/s12863-016-0368-3)

Figure S1. Boxplot of inbreeding coefficient in each generation.

The x-axis represents generation and the y-axis represents inbreeding coefficient.

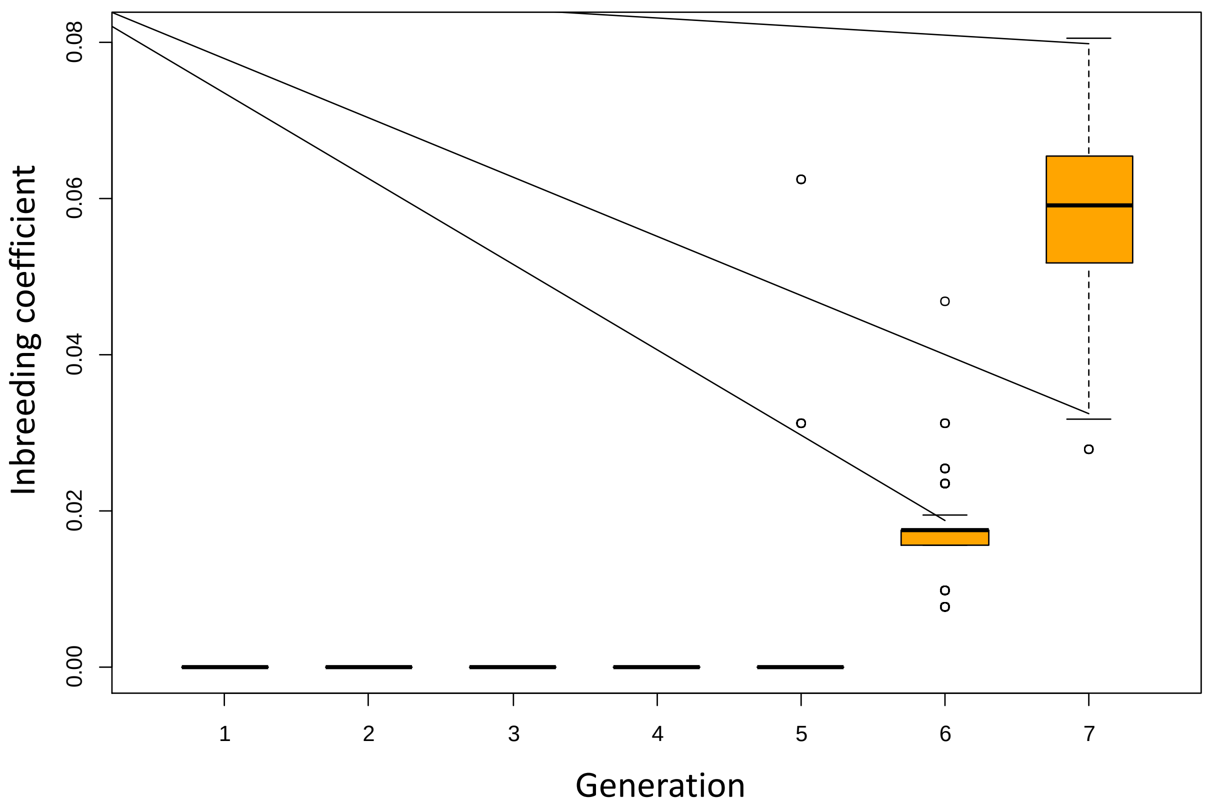

Supplement: Additional file 1: Figure S1. — Boxplot of inbreeding coefficient in each generation. (PDF 167 kb) [file 12863_2016_368_MOESM1_ESM.pdf]
