# Supplementary material for: SNP- and haplotype-based genome-wide association studies for growth, carcass, and meat quality traits in a Duroc multigenerational population
Source: BMC Genet. 2016 Apr 19;17:60. doi: 10.1186/s12863-016-0368-3 (PMC4837538; doi:10.1186/s12863-016-0368-3)

Figure S2. Average linkage disequilibrium coefficient (r^2) values are plotted against intermarker distance for all autosomal chromosomes.

The plot on the right upper corner is the zoomed area of the bigger plot. The x-axis represents the distance between SNPs (kb) and the y-axis represents r^2 between SNPs.

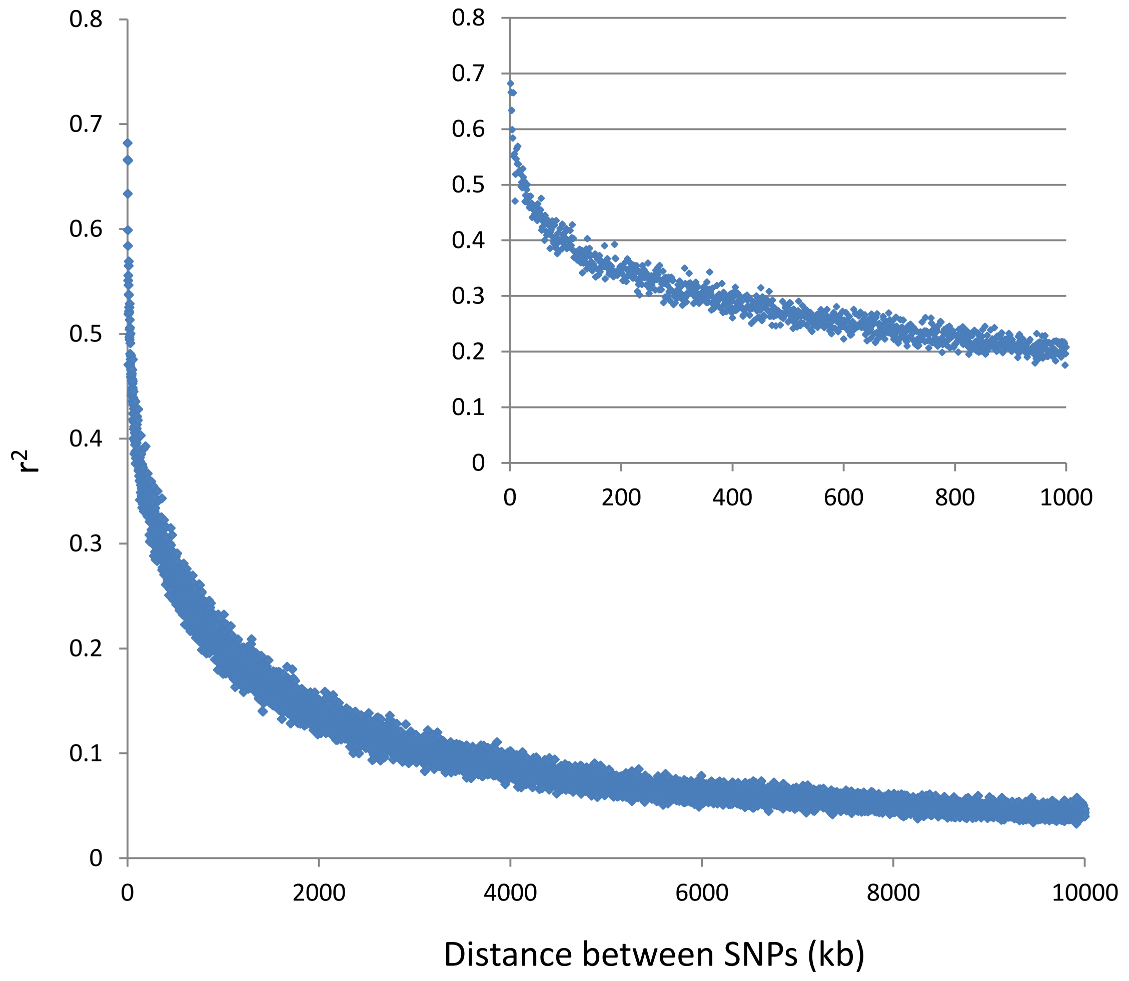

Supplement: Additional file 3: Figure S2. — Average linkage disequilibrium coefficient (r2) values plotted against intermarker distance for all autosomal chromosomes. (PDF 407 kb) [file 12863_2016_368_MOESM3_ESM.pdf]
