# Supplementary material for: SNP- and haplotype-based genome-wide association studies for growth, carcass, and meat quality traits in a Duroc multigenerational population
Source: BMC Genet. 2016 Apr 19;17:60. doi: 10.1186/s12863-016-0368-3 (PMC4837538; doi:10.1186/s12863-016-0368-3)

Figure S3. Quantile-quantile plot of the P-value for SNP-based genome-wide association study in base phenotype.

Once the base phenotype was generated, heritability was estimated to be 0.30 ± 0.06 by using the model to correct the phenotype. We then performed a SNP-based genome-wide association study (GWAS) using this phenotype. Circles represent the observed statistics and the black line represents where the dots are expected to fall under the null hypothesis of no association. The results showed that the simulated phenotype was appropriate to be used as the base phenotype under null hypothesis of no phenotype-SNP correlation.

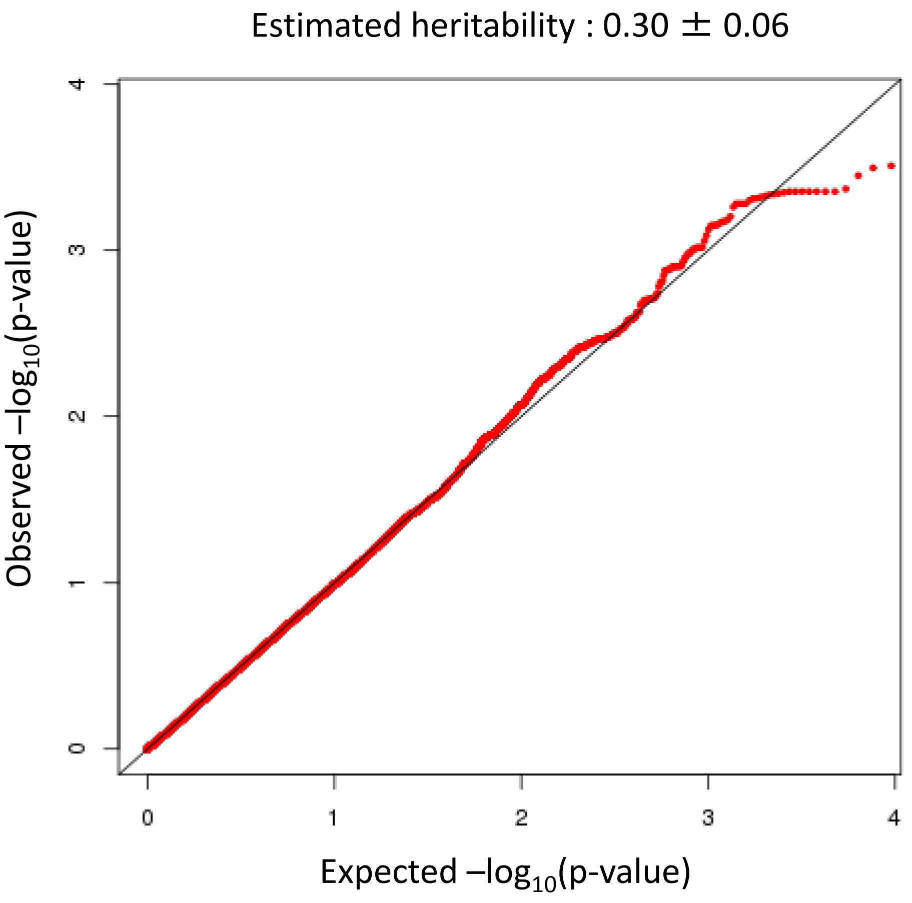

Supplement: Additional file 4: Figure S3. — Quantile-quantile plot of the P-value for SNP-based genome-wide association study in base phenotype. (PDF 279 kb) [file 12863_2016_368_MOESM4_ESM.pdf]
