# Supplementary material for: SNP- and haplotype-based genome-wide association studies for growth, carcass, and meat quality traits in a Duroc multigenerational population
Source: BMC Genet. 2016 Apr 19;17:60. doi: 10.1186/s12863-016-0368-3 (PMC4837538; doi:10.1186/s12863-016-0368-3)

Figure S4. Scoring criteria for feet and leg structure soundness traits.

The scoring criteria is the basis of the desirability of the condition (non-linear scoring method). Front and rear legs are graded ranging from scores of 1 (straight) to 5 (soft).

Front leg | Rear leg

Score 1
no flexibility
straight

Score 2

Score 3
moderate flexibility

Score 4

Score 5
soft

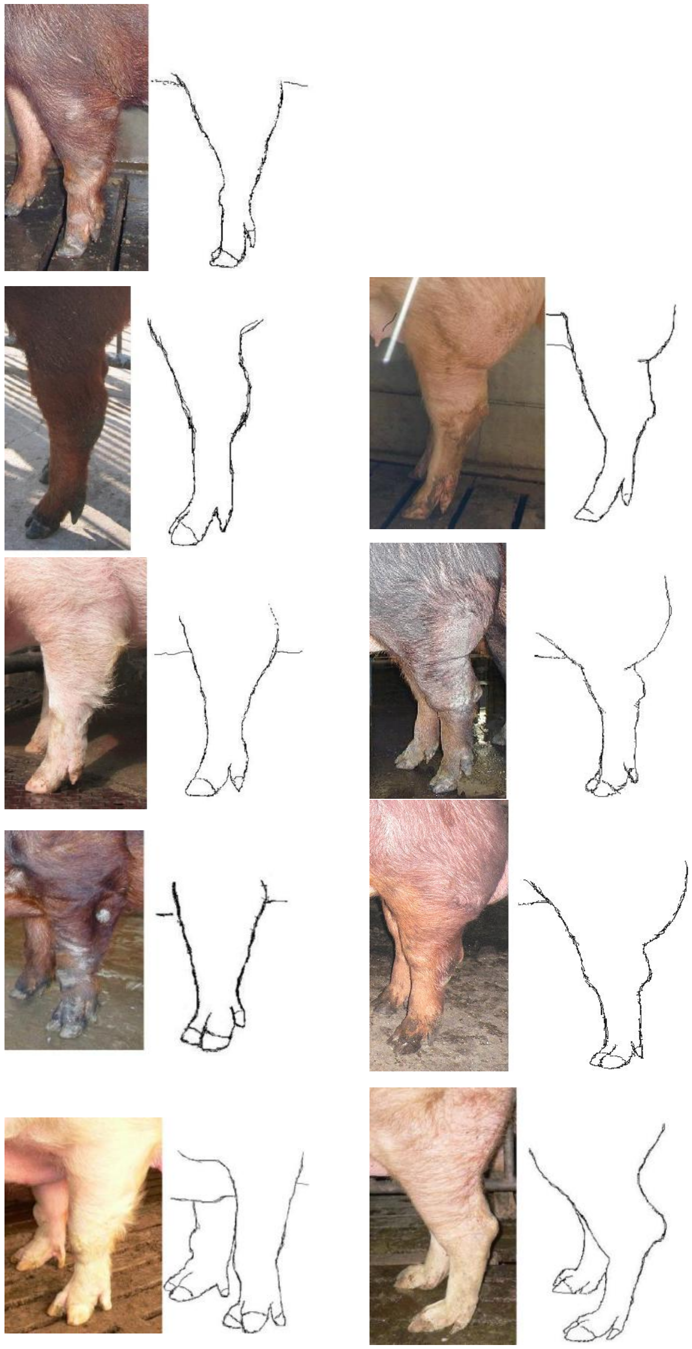

Supplement: Additional file 5: Figure S4. — Scoring criteria for feet and leg structure soundness traits. (PDF 320 kb) [file 12863_2016_368_MOESM5_ESM.pdf]
