# Supplementary material for: SNP- and haplotype-based genome-wide association studies for growth, carcass, and meat quality traits in a Duroc multigenerational population
Source: BMC Genet. 2016 Apr 19;17:60. doi: 10.1186/s12863-016-0368-3 (PMC4837538; doi:10.1186/s12863-016-0368-3)
Supplement: Additional file 6: Figure S5. — Classification of fat accumulation of carcass cross sectional image. (PDF 206 kb) [file 12863_2016_368_MOESM6_ESM.pdf]

Figure S5. Classification of fat accumulation of carcass cross sectional image.

The top panel shows the fat area of carcass cross section at 4-5 rib; and the bottom panel shows the fat area of carcass cross section at the middle.

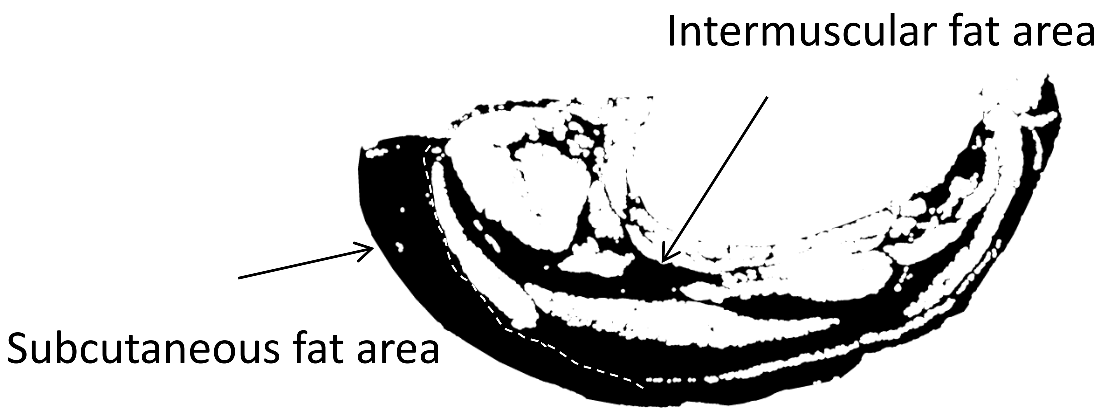

Carcass cross section at 4th -5th rib

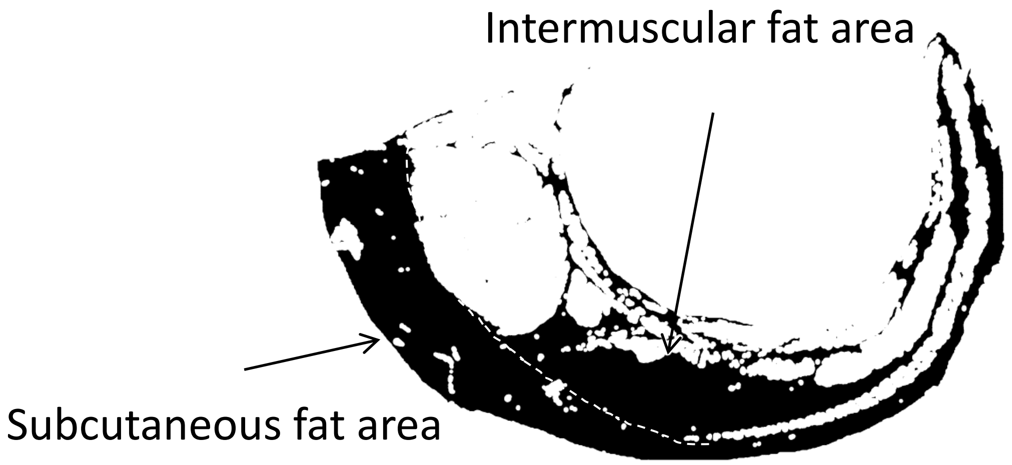

Carcass cross section at the middle
