# Supplementary material for: SNP- and haplotype-based genome-wide association studies for growth, carcass, and meat quality traits in a Duroc multigenerational population
Source: BMC Genet. 2016 Apr 19;17:60. doi: 10.1186/s12863-016-0368-3 (PMC4837538; doi:10.1186/s12863-016-0368-3)

Figure S6. Linkage disequilibrium (LD) of the significant region on chromosome 7.

(A) LD coefficients (r^2) values between 176 SNPs ranged from 34.2 to 42.5 Mb on chromosome 7, and (B) r^2 values between 48 SNPs ranged from 35.0 to 37.5 Mb on chromosome 7. Black fields display r^2 values > 0.80, white and gray fields display r^2 values < 0.80.

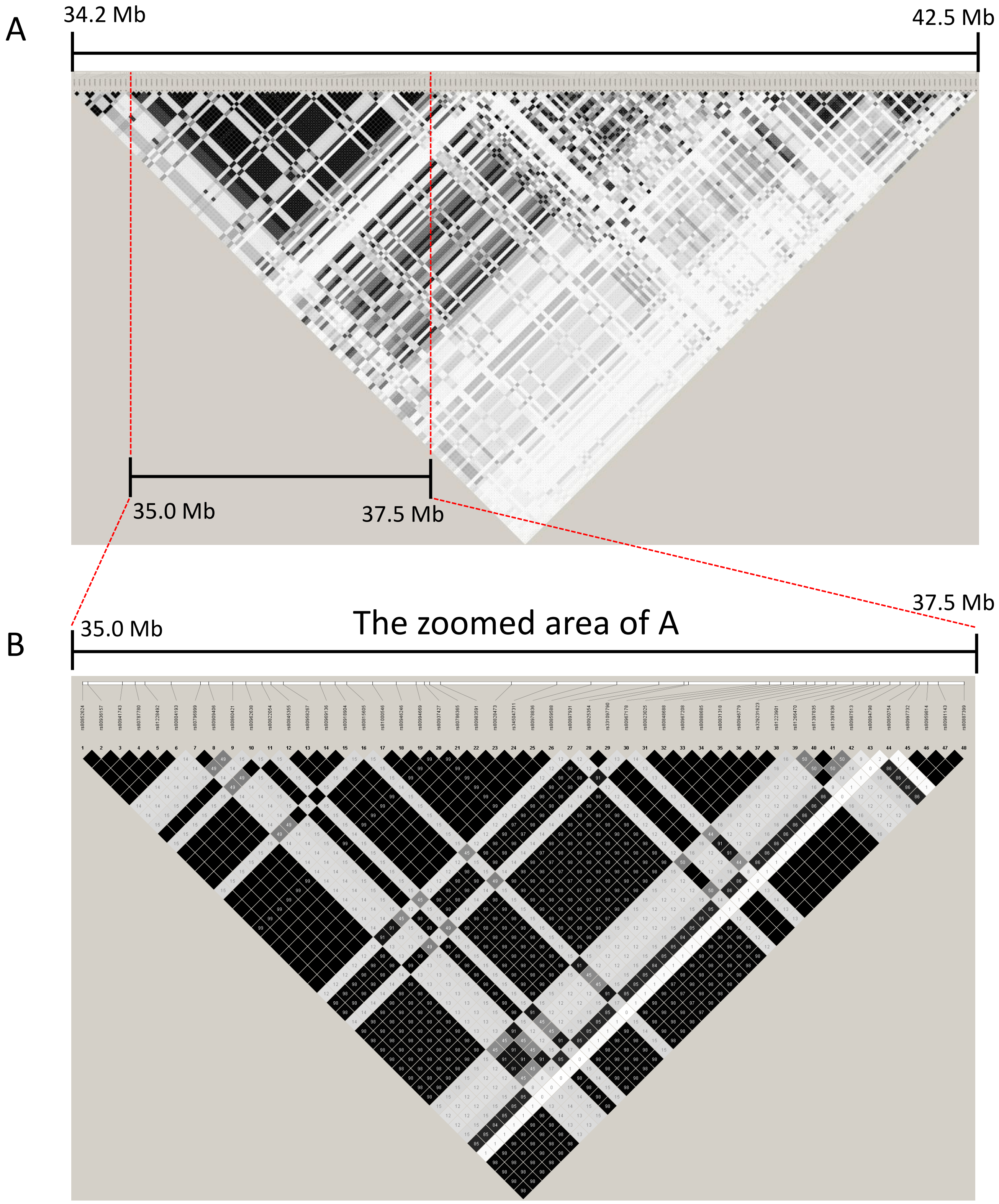

Supplement: Additional file 8: Figure S6. — Linkage disequilibrium (LD) of the significant region on chromosome 7. (PDF 415 kb) [file 12863_2016_368_MOESM8_ESM.pdf]
