# Supplementary material for: SNP- and haplotype-based genome-wide association studies for growth, carcass, and meat quality traits in a Duroc multigenerational population
Source: BMC Genet. 2016 Apr 19;17:60. doi: 10.1186/s12863-016-0368-3 (PMC4837538; doi:10.1186/s12863-016-0368-3)

Figure S7. Comparison of the regional plots by SNP-based and haplotype-based genome-wide association studies (GWAS).

The x-axis represents chromosomal region (Mb) and the y-axis represents $-log_{10}$(p-value). (A) Plots in chromosome 7 (100-110 Mb) for thoracic vertebrae number. (B) Plots in chromosome 7 (40-46 Mb) for all fat area of carcass cross section at the middle. (C) Plots in chromosome 17 (18-24 Mb) for centrifugal water-holding capacity.

A

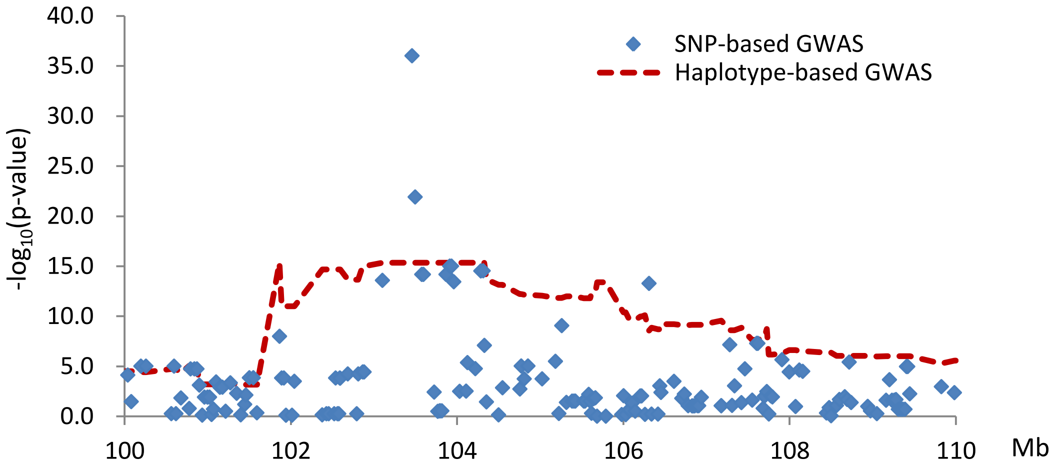

B

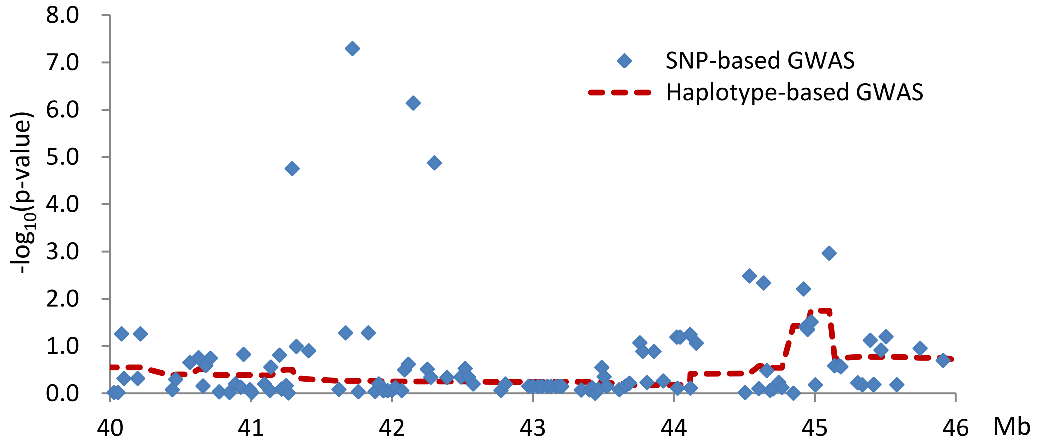

C

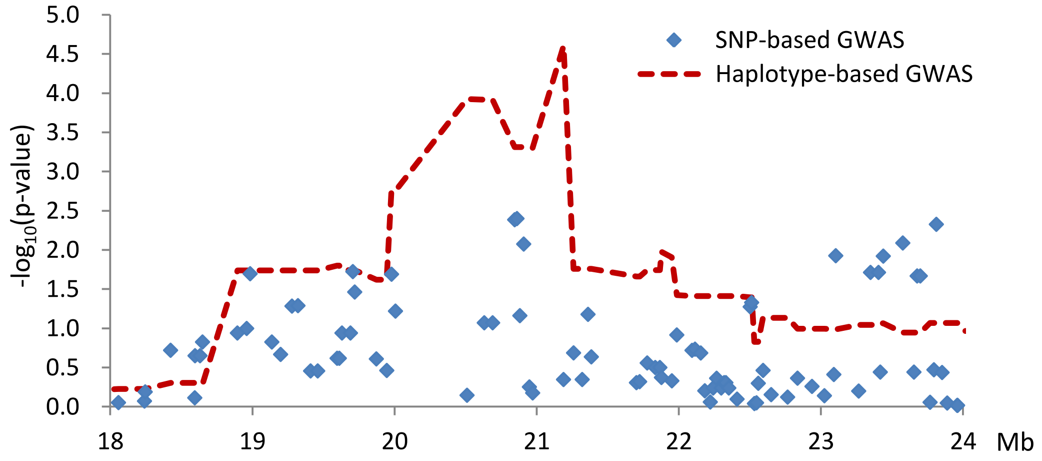

Supplement: Additional file 9: Figure S7. — Comparison of the regional plots by SNP-based and haplotype-based genome-wide association studies (GWAS). (PDF 285 kb) [file 12863_2016_368_MOESM9_ESM.pdf]
